# Urinary nephrospheres indicate recovery from acute kidney injury in renal allograft recipients – a pilot study

**DOI:** 10.1186/s12882-019-1454-3

**Published:** 2019-07-09

**Authors:** Daniela Knafl, Wolfgang Winnicki, Peter Mazal, Ludwig Wagner

**Affiliations:** 1 Division of Nephrology and Dialysis, Department of Medicine III, Medical University of Vienna, Waehringer Guertel 18-20, 1090 Vienna, Austria; 2 Clinical Institute of Pathology, Medical University of Vienna, Waehringer Guertel 18-20, 1090 Vienna, Austria

**Keywords:** Nephrosphere, Acute kidney injury, Kidney transplantation, Tubular regeneration, Organ repair

## Abstract

**Background:**

Acute kidney injury represents a major threat to the transplanted kidney. Nevertheless, these kidneys have the potential to fully recover. Tubular regeneration following acute kidney injury is driven by the regenerative potential of tubular cells originating from a tubular stem cell pool. We investigated urinary sediments of acute kidney injury transplanted patients and compared it to those of non-transplanted patients. Thereby we discovered tubular cell agglomerates, which have not been described in vivo. We hypothesized that these so-called nephrospheres were associated with recovery from acute kidney injury.

**Methods:**

Urine sediment of 45 kidney-transplanted and 19 non-transplanted individuals was investigated. Nephrospheres were isolated and stained for several molecular markers including aquaporin 1 (AQP1) and calcium sensing receptor (CASR). Nephrospheres were cultured to examine their growth behavior in vitro. In addition, quantitative PCR for CASR, AQP1, and podocin (NPHS2) was performed.

**Results:**

Nephrospheres were excreted in the urine of 17 kidney-transplant recipients 7 days after onset of acute kidney injury and were detectable over several days until kidney function was recovered to baseline creatinine levels. None were found in the urine of non-transplanted individuals. Nephrospheres were either AQP1+/CASR+ or AQP1−/CASR+ and could be cultured for 27 days. Mitotic cells could still be visualized after 17 days in culture. Quantitative PCR detected AQP1 in both kidney-transplanted and non-transplanted individuals during the phase of creatinine decline. As a limitation qPCR was only performed for the entire urinary sediment.

**Conclusions:**

Nephrospheres are three dimensional tubular cell agglomerates which appeared in urine of kidney transplant recipients recovering from acute kidney injury. Appearance of nephrospheres in urine was independent of the duration after kidney transplantation. Nephrospheres proliferated in cell culture and kept expressing kidney specific marker. Presence of nephrospheres in urine showed a specificity of 100% and a sensitivity of 60.71% for recovery.

**Electronic supplementary material:**

The online version of this article (10.1186/s12882-019-1454-3) contains supplementary material, which is available to authorized users.

## Background

Kidney failure represents a major human morbidity associated with injuries to the organ, and it is estimated that up to 2 million patients require some form of renal replacement therapy in the industrialized world alone [1]. Although dialysis is considered a reasonable temporary solution to kidney failure, in the long term only kidney transplantation will bring true relief to patients. Indeed, kidney transplantations are a major medical undertaking, with about 20,000 operations taking place in European patients per year [2]. Despite kidney transplantations being routine these days, nevertheless there remains a residual level of uncertainty regarding organ rejection as well as graft loss. Therefore, there is an urgent need to find ways to determine which recipients are on their way to recovery compared to those that might require more concerted efforts to try and reduce the possibility of graft loss.

Acute kidney injury (AKI) represents the consequence of various severe medical conditions such as ischemia; infection; exposure to toxic substances; and obstruction of urine outflow. Independent of the underlying pathogenic mechanism, the topological site of injury is located at the actual site of filtration, i.e. at the tubular cell level. Cellular damage primarily occurs at the proximal tubule, with the outcome of loss of the brush border and disintegration of the tubular cells. Recovery of renal function after AKI involves multiple molecular mechanisms.

Recent literature has described a regenerative potential of renal tubular cells originating from a stem cell pool [3]. Propagation of tubular cells must involve cell replication and migration along the basal membrane to cover areas of cell injury. In vitro studies were able to identify a subpopulation of tubular cells with stem cell-like properties, which are able to form cellular agglomerates [4]. These agglomerates, termed nephrospheres, possess pluripotency and are able to repopulate decellularized kidney scaffolds [5].

It is well known that tubular cells can be detected in urine, using light microscopy and specific protein markers. Such markers include aquaporin1 (AQP1) which is specific for the proximal tubule, and the calcium sensing receptor (CASR) marking the distal tubule. Furthermore, GATA3 and PAX8 are transcription factors, which are specifically expressed in cells of renal tubular origin, and NPHS2, which is a protein involved in the formation of filtration slits of podocytes [6]. Neprilysin (CD10) is a stromal marker protein which is expressed during the entire renal organogenesis in the embryo [7].

The aim of this study was to examine urinary sediments and their components during AKI in kidney transplanted patients compared to non-transplant recipients. For this purpose, we isolated urinary excreted cells and characterized their various properties, such as cellular densities, replicative potential in tissue culture, and expression of kidney-specific marker proteins in the time course of AKI. Thereby we observed remarkable differences between transplant and non-transplant patients. To our best knowledge this is the first study describing nephrospheres in vivo.

## Methods

### Patient recruitment

Patients admitted to the Department of Medicine III, Division of Nephrology and Dialysis of the Medical University of Vienna with AKI were enrolled into this study. AKI was defined according to KDIGO guidelines of the International Society of Nephrology [8]. The Ethics Committee (EC) of the Medical University of Vienna approved the conduct of this study (EC number 1043/2016). All patients gave their informed consent to participate in this study. Clinical data were obtained from detailed chart reviews. Urine collection was performed every 24 h at 7:30 AM. The urine samples were taken from an indwelling urinary catheter and frozen for storage at − 80 °C.

### Immunocytochemistry and Immunohistology

Paraffin-embedded urinary sediment cells were cut in 4 μm sections, deparaffinized and treated as described earlier. In brief, following tissue antigen unmasking in citrate buffer, slides were washed in phosphate buffered saline (PBS) and incubated with the primary antibody. Following two washing steps with PBS the secondary antibody was applied and incubated for one hour at room temperature. After a second washing step, the horseradish peroxidase (HRP) substrate/chromogen mixture was applied under microscopic observation and left until a visible color-reaction had developed. Counterstaining was performed using Mayer’s hematoxylin.

### Immunofluorescence microscopy

Cytopreparations were fixed in acetone for 10 min. The primary antibody (either mouse monoclonal, mAb, or rabbit monospecific polyclonal) was applied and slides were incubated overnight. The next day, the secondary antibody (goat-anti-mouse, Alexa546/goat-anti-rabbit, Alexa488) was applied and after a final PBS washing step, 40 μl of DAPI solution was added onto the cell preparation. Slides were mounted in Vectashield® mounting medium for fluorescence (Vector® Laboratories, Inc. Burlingame, CA) under a coverslip. Antibodies used were AQP1 (made in rabbit) from Merck MilliporeTM, CASR (mAb) from ThermoFisher® Scientific and podocalyxin-like protein 1 (PODXL) (mAb) from the Clinical Institute of Pathology of the Medical University of Vienna.

### Culture of Nephrospheres

Cellular agglomerates were isolated from urine. After centrifugation of urine the pallet was suspended in culture medium. This was layered on a Ficoll-Paque™ Plus gradient and centrifuged for 20 min in a HettichTM Rotanta 46 RSC centrifuge. The interface was then removed, washed in culture medium and seeded into a 12-well plate. Every third day the culture medium was replaced by RPMI-1640 containing 10% FCS and renal tubular cell culture hybrid mix (1:1) [9]. The seventh day the cell layer was passaged using trypsin to liberate the adherently growing cells from the tissue culture surface. Individual cell clones were picked under microscopic observation during trypsin treatment when just liberating from the surface. In a second isolation step, nephrospheres were handpicked with a micropipette under invert microscopic observation. The isolates were suspended in culture medium for incubation and further propagation, in order to initiate tissue culture. Alternatively, an aliquot of the resultant cell suspension was applied to the funnel of the cytocentrifuge (Shandon, UK) and centrifuged onto microscopic slides.

### RNA extraction

One hundred mg of normal kidney tissue obtained from tumor nephrectomy were minced in 1000 μl TriFastTM using the peqlab® mincing device (peqlab® Biotechnologie GmbH, Erlangen, Germany). Furthermore, the urine sediment pallet was resuspended in 50 μl urine which was then lysed in 1000 μl TriFastTM. RNA extraction was performed as described in the company’s manual.

### Quantitative PCR (qPCR)

cDNA was amplified using AQP1, CASR, and NPHS2 specific probes from TaqMan (Thermo Fisher Scientific®) and 2x TaqMan Universal Master Mix in a StepOnePlus qPCR machine (Applied Biosystems®). GAPDH was used as a housekeeping gene to normalize the individual expression levels of AQP1 and CASR regarding the cycle threshold (Ct) [10]. The individual sample gene expression level was normalized to human kidneys obtained from tumor nephrectomy.

### Statistical analysis

Adherence to a Gaussian distribution was determined using the Kolmogorov-Smirnov test. Normally distributed data were described as means ± standard deviations. In case of skewed distribution data were described as medians (25th and 75th percentiles). Qualitative variables were described with counts and percentages and compared using Fisher’s exact test. A two-tailed *p*-value of 0.05 was considered statistical significant. Data were analyzed with SPSS® Statistics (Version 21 for Mac).

### Limitations

Unfortunately, we were not able to obtain daily follow-up urine samples from all patients, which represents a limitation of this study. Furthermore, qPCR was performed for the entire urinary sediment specimen and not for nephrospheres only. In addition, three urinary sediment samples of the transplanted group were not investigated with qPCR.

## Results

### Urinary sediments of Allografted AKI recipients contain Nephrospheres

To determine whether urinary sediments of AKI patients with allografted kidneys differed from those patients who were no kidney transplant recipients, samples were collected from 45 kidney allograft recipients. Urine collection was undertaken over time, from initial AKI diagnosis until kidney function was restored, or renal replacement therapy was established. Following centrifugation, sediments were recovered and examined microscopically by staining as well as a variety of immunofluorescence methods. Apoptosis was examined using light microscopy. As a control, sediments of 19 non-allograft AKI control patients were also analyzed.

**Fig. 1:**
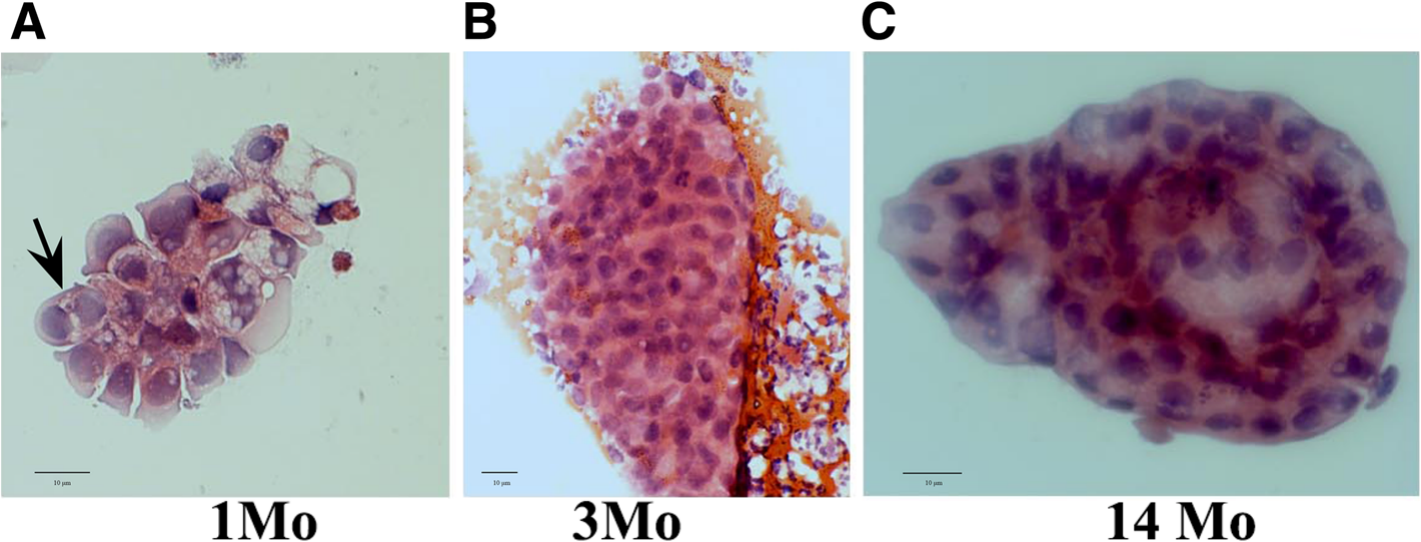
Hematoxylin/eosin staining of urinary nephrospheres from renal allotransplant recipients. Cellular agglomerates of a patient undergoing AKI one month after renal transplantation, the arrow indicates a mitotic cell (**a**), large cellular agglomerate of a patient undergoing AKI three months after transplantation (**b**), and from a patient 14 months after transplantation (**c**)

**Fig. 2:**
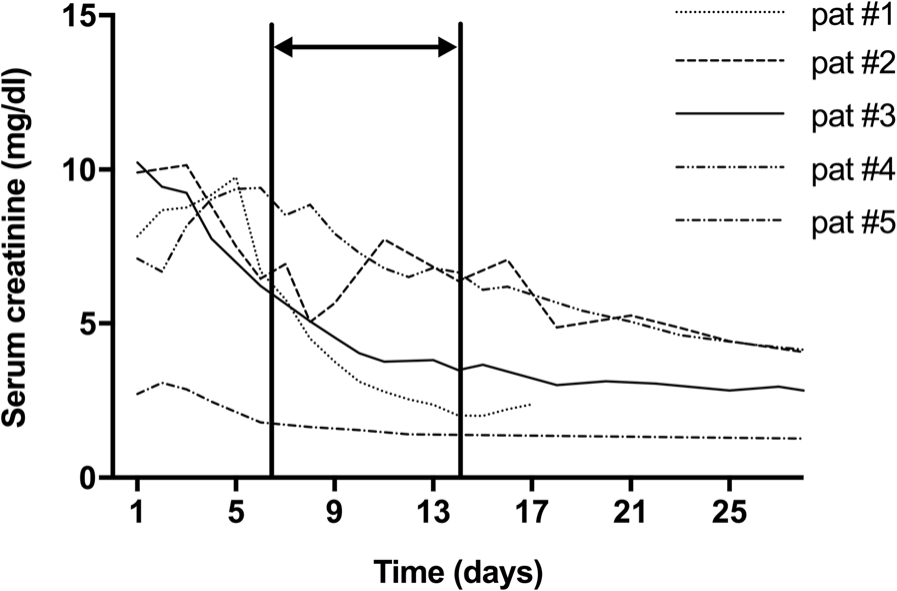
Period of appearance of nephrospheres in urine following AKI. Serum creatinine kinetics of five renal transplant recipients over time. Nephrospheres were observed during the decline of serum creatinine in a time frame of 7 to 14 days. This period is indicated by an arrow

The results of hematoxylin/eosin staining revealed multilayered, three-dimensional cell agglomerates, comprising between ten to more than 100 cells, in 17 of the allograft recipients (Table 1). These cells contained vacuoles and infrequently underwent mitosis (Fig. 1). These spherical structures, herewith termed nephrospheres, were observed both during the period of serum creatinine decrease characteristic of AKI as well as at the time of renal function regain. Appearance of nephrospheres in urine was independent of the duration after kidney transplantation. These nephrospheres could be observed seven days after onset of AKI and over a period of seven days, depending on the individual patient, during improvement of kidney function (Fig. 2). Based on light microscopic examination, it was possible to identify the cells comprising the nephrospheres as tubular cells, albeit their exact source along the nephron remained to be determined. While tubular cells were found in the urine of both transplanted and non-transplanted AKI patients, nephrospheres were exclusively observed in the transplanted group. All patients who excreted nephrospheres in urine recovered from AKI (*n* = 17), while only 39.29% (*n* = 11) of kidney transplant patients who did not exhibit nephrospheres in urine (*n* = 28) recovered. Thereby presence of nephrospheres in urine showed a sensitivity of 60.71% and a specificity of 100% for recovery from AKI with a positive predictive value (PPV) of 100% and a negative predictive value (NPV) of 60.71%.

**Table 1:** Demographics and laboratory values. The degree of presence of nephrospheres in urine was determined according to observations of two independent examiners. Estimated glomerular filtration rate (eGFR), blood urea nitrogen (BUN), C-reactive protein (CRP), acute kidney injury (AKI), atypical hemolytic uremic syndrome (aHUS), thrombotic microangiopathy (TMA)

	Transplanted (*n* = 45)	Non-transplanted (*n* = 19)	*p*-value
Female—no. (%)	14 (31.12)	11 (57.89)	*p* = 0.055
Age [years]	59.02 ± 13.51	58.95 ± 21.70	*p* = 0.988
Serum Creatinine [mg/dL]	4.152 ± 2.460	3.749 ± 1.635	*p* = 0.516
eGFR [ml/min/1.73m^2^]	23.76 ± 17.98	19.71 ± 11.33	*p* = 0.368
BUN [mg/dl]	45.667 ± 27.654	51.818 ± 18.331	*p* = 0.378
CRP [mg/dl]	4.395 (1.052;9.46)*	5.190 (0.84;10.98)*	*p* = 0.371
Degree of presence of nephrospheres in urine—no. (%)			
None	28 (62.2)	19 (100)	***p*** **< 0.001**
Low	9 (20.0)	0 (0)	***p*** **= 0.048**
Medium	4 (8.9)	0 (0)	*p* = 0.309
High	4 (8.9)	0 (0)	p = 0.309
Reasons for AKI—no. (%)			
Infection	21 (46.7)	8 (42.1)	*p* = 0.789
Ischemia	10 (22.2)	3 (15.8)	*p* = 0.738
Chemotherapy	2 (4.4)	4 (21.1)	*p* = 0.058
Heart failure	5 (11.1)	3 (15.8)	*p* = 0.685
aHUS/TMA	3 (6.7)	1 (5.3)	p = 0.999
Graft rejection	3 (6.7)	0 (0)	*p* = 0.548
Postrenal causes	1 (2.2)	0 (0)	p = 0.999

* = median (upper quartile;lower quartile); numbers in bold represent statistical significant values

**Fig. 3:**
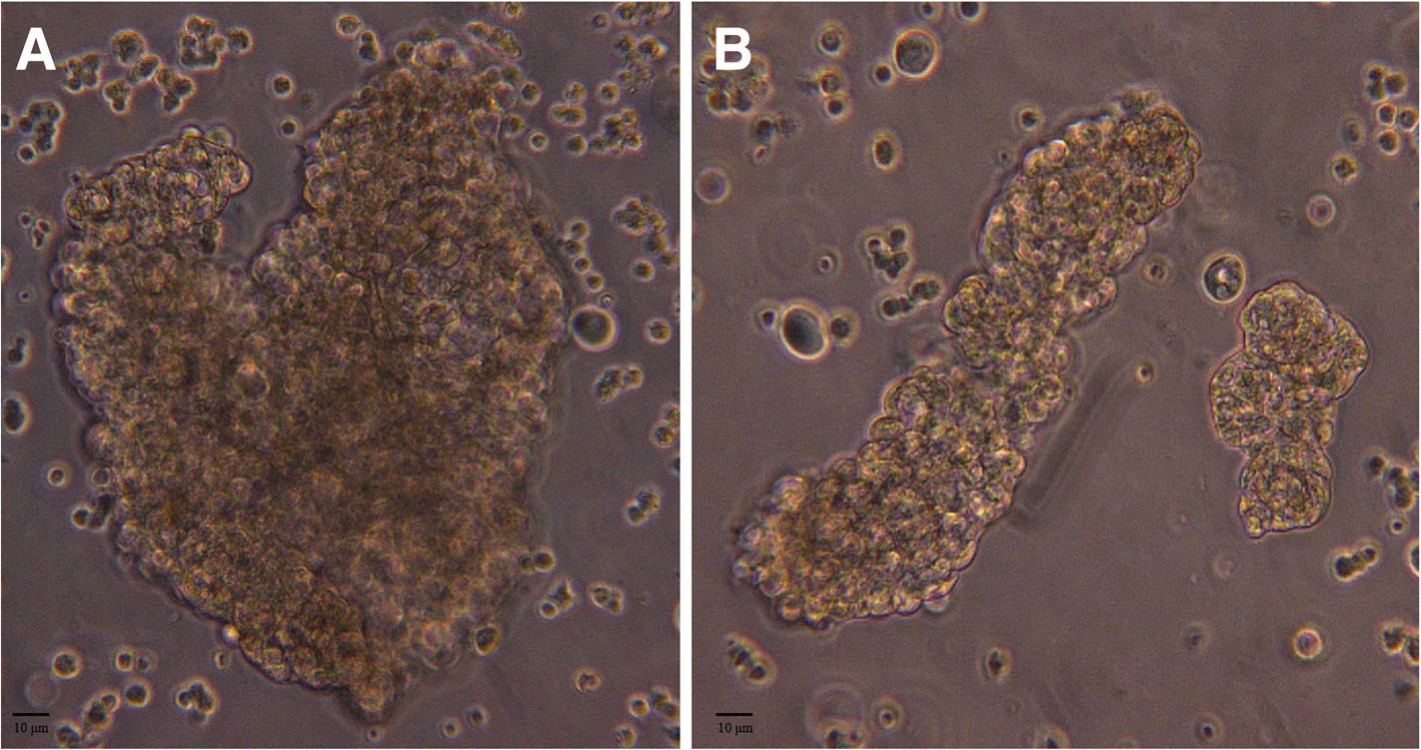
Phase contrast microscopy of freshly isolated nephrospheres. Handpicked nephrosphere under phase contrast (**a**). Rod-shaped cellular agglomerate, which most likely represents a tubular cast (**b**)

In contrast, sediments from control patients contained mostly apoptotic tubular cells displaying features such as nuclear condensation, fragmentation, membrane lysis, and expulsion of the nucleus. Some of the tubular cells were found in groups of four to five cells, but these groups differed markedly from the agglomerates seen in transplant recipients (Fig. 3).

Therefore, it would seem that nephrospheres might distinguish between healing transplanted AKI patients and otherwise not transplanted ones, and it would be useful to be able to expose the structures’ biological source along the nephrons.

### Nephrospheres originate from the proximal and distal tubule and express CD10

To determine the precise origin of the tubule cells incorporated into nephrospheres, immunofluorescence staining was performed using commercially available marker proteins AQP1, indicative for the proximal tubule, and CASR specific for the distal tubule. In addition, staining was also undertaken for PODXL, a protein specific for podocytes forming the kidneys’ filtration apparatus. Furthermore, immunohistology staining was also performed for the endometrial stroma marker CD10, which is a relevant protein in renal embryogenesis, and GATA3 and PAX8, which are kidney-specific transcription factors [11]. Standard immunofluorescence staining controls were applied, including the use of tubular urinary sediment cells as positive controls for AQP1 and CASR.

**Fig. 4:**
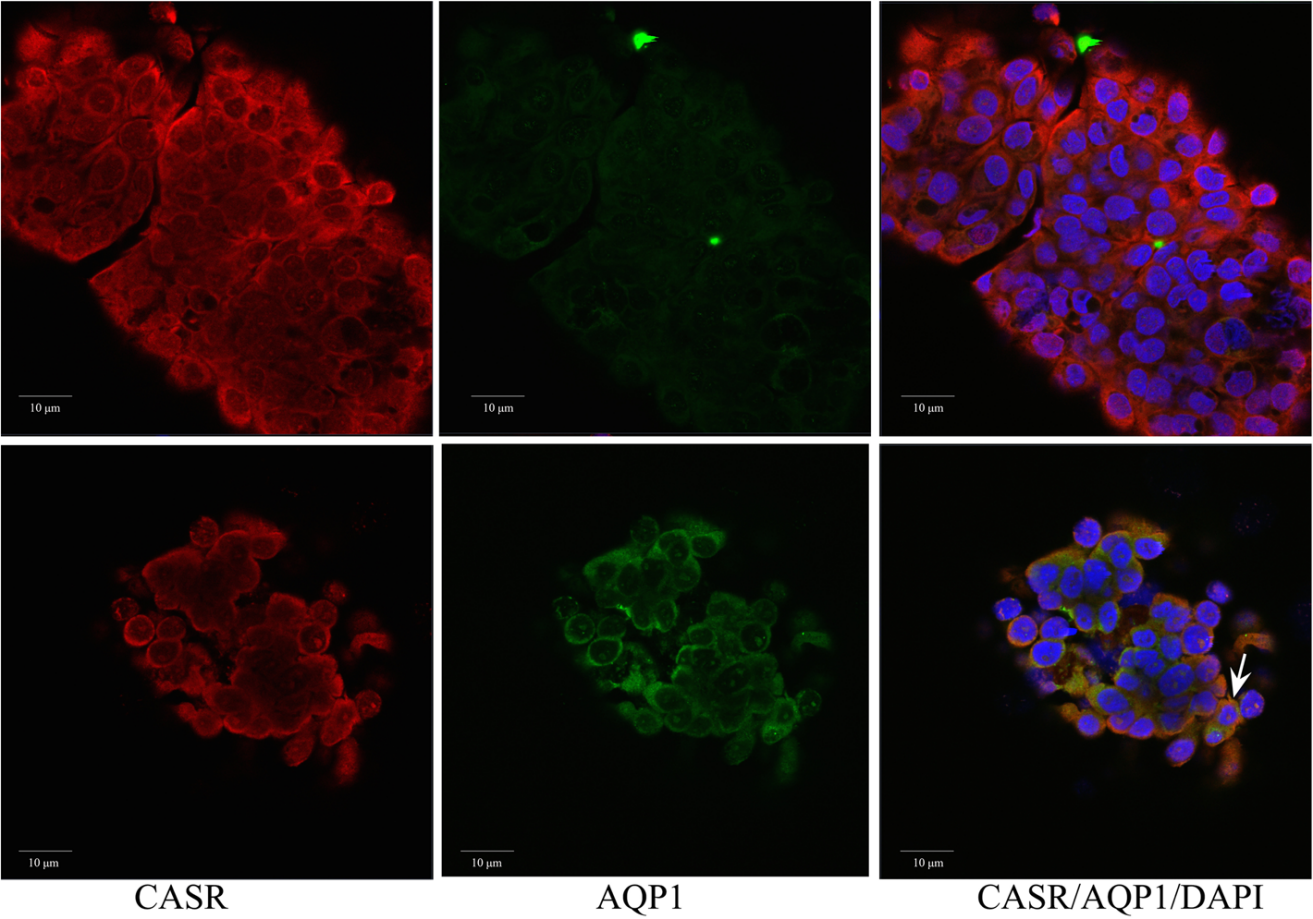
Dual color confocal microscopy of cytospin preparations of freshly isolated nephrospheres. Depicted are nephrospheres from a patient with the phenotype AQP1−/CASR+ (upper panel) and a patient with the phenotype AQP1+/CASR+ (lower panel). A mitotic structure is indicated by an arrow. AQP1 expression is indicated as green, CASR expression as red, DAPI is blue

The results showed that CASR (red) expression was present on almost all nephrospheres but varied in its intensity (Fig. 4). Furthermore, levels of AQP1 (green) expression varied between the spheres and among the cells therein (Fig. 4). Staining for the podocyte marker PODXL was positive in only one patient. As indicated in Table 2, CASR+ nephrospheres additionally displaying an AQP1- phenotype were more frequent than AQP1+ agglomerates, indicating that slightly more cells contained within nephrospheres emerged from the distal tubule. The nephrosphere-phenotype was consistent for each patient. Paraffin-embedded nephrospheres were positive for the highly reliable immunohistochemical marker CD10 as well as for GATA3 and PAX8 (Additional file 1: Figure S1) [7].

**Table 2:** Immunofluorescent staining of AQP1, CASR and PODXL of nephrospheres. Expression of AQP1, CASR and PODXL in nephrospheres from urine of kidney-transplant recipients is given as negative (−), positive (++) and highly positive (++)

ID	AQP1	CASR	PODXL
1	++	++	–
2	–	+	–
3	–	+	–
4	+ −	++	–
5	+	+	–
6	+	+	–
8	++	+	–
9	++	+	–
10	++	–	–
11	+	++	–
12	–	–	+
13	+	+	–

Hence, since the majority of nephrospheres were decorated with CASR and less so by AQP1, it would be reasonable to assume that they originated from the distal tubule or that distal tubule cells covered proximal tubule cells situated within the nephrosphere. Moreover, the presence of the markers CD10, GATA3, and PAX8, hinted to an embryogenic potential for these kidney-borne cells, and it would be interesting to determine whether these structures had any proliferative potential and could be propagated in vitro for further studies.

### Nephrospheres grow and expand in tissue culture and form new clones

As recent data has shown, in vitro engineered nephrospheres possess the ability to expand and repopulate kidney scaffolds and, therefore, we thought it would be interesting to examine whether patient-derived nephrospheres could grow under in vitro conditions. Hence, isolated nephrospheres were seeded in culture using renal tubular cell culture mix and observed by invert microscopy.

**Fig. 5:**
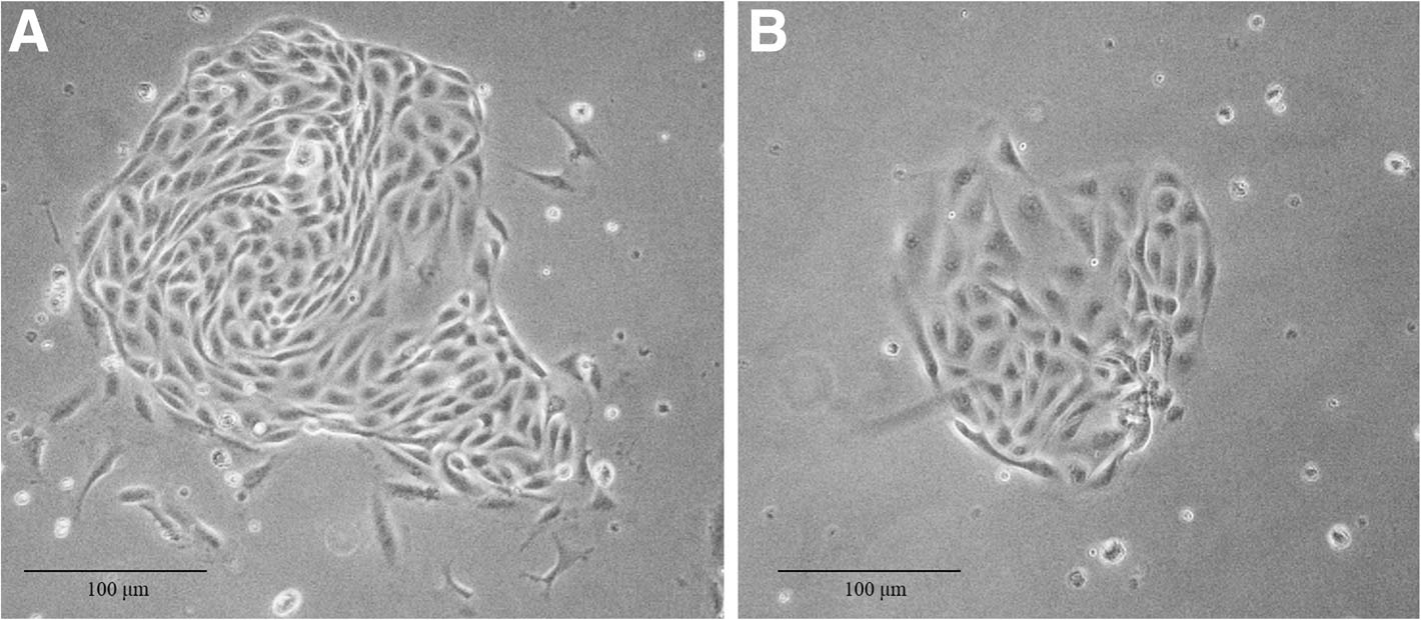
Tissue culture of isolated nephrospheres. Isolated nephrospheres in tissue culture depicted by phase contrast microscopy showed migrating cells distributing off the site of adherence and proliferation (**a**) or expanding growth towards the site of adherence (**b**)

When first put into culture, these freshly isolated cell agglomerates either displayed rod-like or tubular structures (Fig. 3). After two to three days in culture nephrosphere cells started to adhere to the surface of the culture plate. Four to five days in culture thereafter, individual new clones started to form (Fig. 5). In the following days, these clones were observed to be further expanding at varying growth rates, displaying morphological diversity. These freshly formed clones were eventually isolated and transferred into fresh media within new wells, where about 3% kept expanding, and could be passaged for a third and fourth time, without showing senescence or a growth stop even after 27 days in culture.

**Fig. 6:**
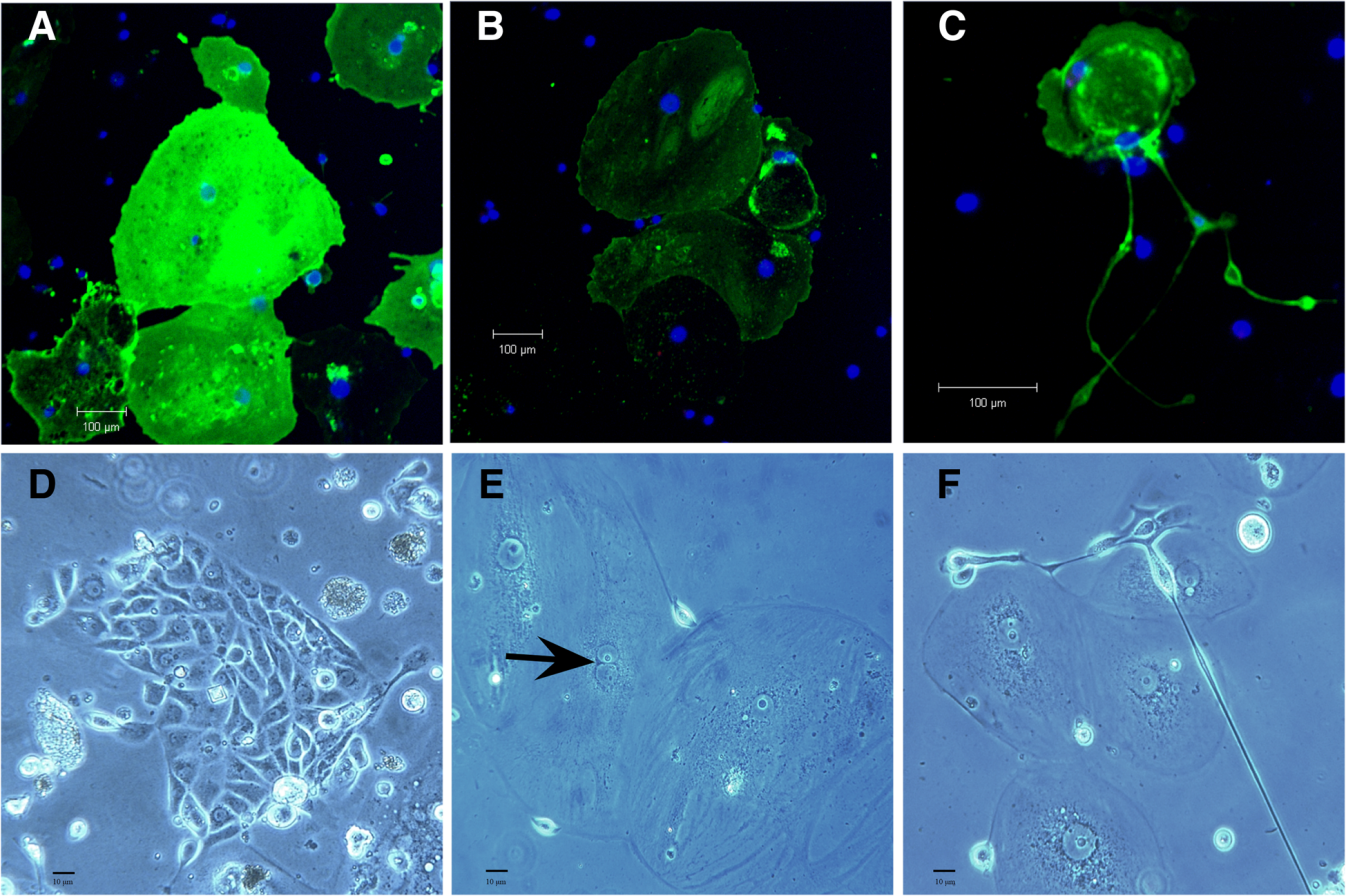
In situ confocal immunofluorescence and phase contrast microscopy of tissue culture grown nephrospheres. In bulk-grown nephrospheres after 20 days in tissue culture exhibited various intensity of AQP1 expression (green) and variability in cell size. AQP1+ cells covered the plate surface and were juxtaposed to AQP1- cells (**a**, **b**) and formed cell-to-cell contact. Infrequently, AQP1+ cells showed long dendritiform, podocytic extrusions (**c**). Proliferative foci between spreading cells were visualized by phase contrast microscopy (**d**) with mitotic cells (→ telophase) (**e**) and cells with podocytic extrusions (**f**)

As a result the morphology of these adherent cells varied from those covering large surface areas to rounded cell bodies extending long pseudopodic structures, also above the basal layer of other cells (Fig. 6 upper panel). Phase contrast observation of bulk cultures of nephrospheres showed proliferative foci (Fig. 6d) among cells covering large areas of which some displayed mitotic features (Fig. 6e) and extending ruffles (Fig. 6f), indicative of cells forming contact with neighboring ones. After 27 days in culture, cells started to show signs of senescence with developing vacuoles and loss of cell adhesion.

Hence, nephrospheres appeared to possess at least some regenerative powers, and it seemed logical to investigate whether they had the ability to form kidney tubules.

### Newly formed Nephrosphere clones show affiliation to kidney tubules

To determine if newly formed nephrospheres were still expressing marker proteins specific for kidney tubular cells, freshly formed nephrospheres were in situ stained for AQP1, CASR, DAPI and PODXL after 20 days in bulk culture.

All newly grown agglomerates demonstrated AQP1 (green) staining in various intensities, going from very high (Fig. 6a) to moderate intensities (Fig. 6b). AQP1+ cells could be observed covering the plate and were directly growing along AQP1- cells, with which they were forming cell-to-cell contact (Fig. 6a, b). Moreover AQP1+ cells could be depicted developing long dendritiform, podocytic extrusions (Fig. 6c). These cells started to grow proliferatively (Fig. 6d), exhibiting cell division indicated by mitotic cells, which were found in telophase (Fig. 6e) and cells with podocytic extrusions (Fig. 6f).

Thus it appears that nephrospheres grown in bulk culture over 20 days still expressed marker proteins specific to the proximal kidney tubule and thereby still belonged to functional renal cells. The cells ability to grow, form podocytic protrusions and undergo mitosis indicated that cells from nephrospheres still possess potential to grow and probably differentiate.

In addition it was considered important to express and compare the overall representation of kidney tubular proteins in transplanted to non-transplanted AKI patients.

### Both transplanted and non-transplanted AKI patients show more proximal than distal tubular cells in overall urinary sediment

In order to establish quantitative data about presence of tubular cells and regenerative glomerular cells among urinary sediment cells, qPCR for AQP1, CASR, and NPHS2 were performed from urinary sediment of non-transplant patients (*n* = 19) and transplanted patients (*n* = 42). Normal human kidney tissue was used as reference (Additional file 1: Figure S2). Individual patient results of AQP1, CASR, and NPHS2 are presented as relative expression level in comparison to normal human renal tissue. In order to calculate AQP1, CASR, and NPHS2 expression in sediment cells GAPDH expression was chosen as reference.

Overall 72.13% of patients showed positive AQP1 expression, indicative for proximal tubule cells, in urinary sediment, which was approximately the same in non-transplant recipients (73.68%) as in transplant patients (71.43%). This did not reach statistical significance due to broad spreading of the values (*p* = 0.246).

In a further attempt to focus on different tubular stretches of the nephron we investigated CASR mRNA expression in urinary sediment, which would indicate presence of tubular cells further distal in the nephron. In general CASR positive cells were found in urine of 32.79% of patients. 21.05% of non-transplanted patients and 38.09% of transplanted patients revealed CASR positive cells in urine. There was no significant difference between transplanted and non-transplanted patients, although expression levels were overall higher in transplanted individuals (*p* = 0.999).

In summary, the extent of CASR mRNA expression was much lower when compared to the extent of AQP1 expression. This indicated that mRNA of distal tubular cells were less frequently found in the urinary sediment of transplanted and non-transplanted AKI patients than those originating from the proximal tubule. No topographical differences of tubular cell origin in urinary sediments between transplanted and non-transplanted AKI patients could be demonstrated via qPCR.

## Discussion

Patients receiving donor kidneys have a certain risk of transplant failure due to a number of reasons, including acute kidney injury. Hence, there is urgency in ascertaining whether transplantations are successful, and for this purpose developing relevant markers would be very useful. One such indicator is represented by nephrospheres, which are agglomerations of cells derived from the kidney tubules. Although these structures have been engineered previously in the context of in vitro organogenesis (see below), here we could confirm for the first time their exclusive presence in the urine of kidney transplant recipients following AKI, mostly on their way to recovery. In addition, based on nephrosphere expression of AQP1 and CASR, we were also able to determine their origin, namely the proximal and distal tubule. Moreover, these structures could grow in tissue culture and develop new clones, which latter could affiliate with kidney tubules. Finally, we could show that nephrosphere development was not a function of the number of tubular cells excreted by patients into urine.

In reference to the appearance of nephrospheres, these consisted of renal progenitor cells in the urine of kidney-transplanted individuals following ischemic or noxious kidney injury, seven to 14 days after peak levels of serum creatinine. Regarding their expression of kidney-specific marker proteins, such as the aforementioned proximal tubular marker AQP1 and the tubular cell-specific marker CASR, this served to recapitulate that they did not originate from urinary epithelia as they differed in both morphology and surface antigen expression. Based on microscopic observations, nephrospheres had the potential to proliferate by undergoing mitosis, thereby indicating tubular injury repair [5, 12–14].

As mentioned above, nephrospheres have only recently been known in the literature from in vitro experimental procedures using disintegrated tubular cells of embryonic or adult kidneys, but have never been described in vivo [4, 5]. In laboratory investigations, agglomerates of renal stem and -progenitor cells exhibit the ability to self-renew, differentiate into various cell lineages, and survive in vivo in an undifferentiated state [4]. Recent data suggest that nephrospheres could be a good tool for scaffold repopulation, thereby representing a milestone on the way to experimental whole-kidney reconstruction [5].

Nevertheless, the question arises as to why these often large nephrospheres can be found in kidney transplanted but not in non-transplanted individuals with AKI. There are three hypotheses: (i) Since transplantees require immunosuppression medication, this will tend to make them more susceptible to viral infection and increased virulence. It is well known that certain viruses facilitate or stimulate cell proliferation and oncogenesis [15]. An epitheliotropic virus may have the potential to transform tubular cells, leading them to proliferate excessively so as to form nephrospheres. (ii) The recipient’s donor-specific antibodies (DSAs) attach to cell membranes of tubular cells by recognizing a non-HLA associated donor-specific protein variant. This initiates an intracellular cascade, eventually leading to cell stimulation and a proliferative response. The Colton blood group antigen of AQP1 may just be one example of a non-HLA donor recipient mismatch locus. (iii) Kidneys destined for transplantation undergo ischemic conditions prior to surgery, whereby ischemia is a well-known stimulus for cell proliferation and angiogenesis. Hence, these organs are preconditioned for shedding tubular cells for the purpose of repair. We consider the third possibility as the most like one.

AKI is usually not investigated by kidney biopsy. However, AKI is a prevalent condition in kidney-transplanted individuals, with numerous potential inciting causes associated with significant risk for poor long-term graft outcomes. Urinary sediments are easily obtainable specimens, which when evaluated for nephrosphere cells, can serve as an indicator for both organ recovery and kidney repair, as well as a key object of investigation for further analyses.

## Conclusion

Nephrospheres represent structures, consisting of tubular cell agglomerates which possess the ability to proliferate and undergo mitosis. Thereby nephrospheres still expressed specific kidney tubule marker proteins. Nephrospheres were excreted in urine of kidney transplant recipients undergoing AKI, however they were not found in non-transplant AKI patients. Presence of nephrospheres in urine reflected tubular regeneration of the transplanted kidney and was associated with a 100% chance for recovery from AKI. The absence of nephrospheres was associated with 61% probability of failure to recover from AKI. This is the first report of the presence of nephrospheres in urine, which have up to now only been described in in vitro experiments. Future studies are needed to determine the origin and triggers for nephrosphere-formation.

## Additional file

Additional file 1: **Figure S1.** Immunohistochemical staining of urinary nephrospheres. Staining for cytokeratin 7 (CK7) was positive for all nephrospheres. The extent of neprilysin (CD10) staining varied strongly among individual cells within different nephrospheres. GATA3 and PAX8 were positive. **Figure S2.** Quantitative PCR of urinary sediment cells for AQP1 and CASR expression. Relative CASR expression (red bar), AQP1 expression (green bar), and NPHS2 expression (blue bar) in urinary sediment cells. Numbers 2–20 represent urinary cells from non-transplanted patients undergoing AKI; Numbers 21–62 represent urinary cells from transplant recipients undergoing AKI. Values represent fold expression relative to normal healthy kidney tissue (sample 1). (DOCX 4362 kb)

## Data Availability

The datasets generated and analysed during the current study are available from the corresponding author on reasonable request.

## Abbreviations

AKI: Acute kidney injury
AQP1: Aquaporin 1
CA: California
CASR: Calcium sensing receptor
CD10: Nephrilysin
CM: Culure medium
DAPI: 4′,6 diamidin-2-phenylindol
DSA: Donor-specific antibody
EC: Ethics committee
GAPDH: Glycerinaldehyd-3-phosphat-dehydrogenase
GATA3: GATA binding protein 3
HLA: Human leukocyte antigen
HRP: Horseradish peroxidase
KDIGO: Kidney Disease Improving Global Outcomes
mAb: monoclonal antibody
mRNA: messenger ribonucleic acid
NPHS2: Podocin
PAX8: Paired box 8
PBS: Phosphate buffered saline
PCR: Polymerase chain reaction
PODXL: Podocalyxin like
qPCR: quantitative polymerase chain reaction
RNA: ribonucleic acid

## References

[1] Peters F, Westphal C, Kramer A, Westerman R (2018). Is the rise in the prevalence of renal replacement therapy at older ages the Price for living longer?. Front Public Health.

[2] Ostermann M, Chang RW (2007). Acute kidney injury in the intensive care unit according to RIFLE. Crit Care Med.

[3] Romagnani P (2013). Of mice and men: the riddle of tubular regeneration. J Pathol.

[4] Bombelli S, Zipeto MA, Torsello B, Bovo G, Di Stefano V, Bugarin C, Zordan P, Vigano P, Cattoretti G, Strada G (2013). PKH(high) cells within clonal human nephrospheres provide a purified adult renal stem cell population. Stem Cell Res.

[5] Bombelli S, Meregalli C, Scalia C, Bovo G, Torsello B, De Marco S, Cadamuro M, Vigano P, Strada G, Cattoretti G (2018). Nephrosphere-derived cells are induced to multilineage differentiation when cultured on human Decellularized kidney scaffolds. Am J Pathol.

[6] Mantilla JG, Antic T, Tretiakova M (2017). GATA3 as a valuable marker to distinguish clear cell papillary renal cell carcinomas from morphologic mimics. Hum Pathol.

[7] McCluggage WG, Sumathi VP, Maxwell P (2001). CD10 is a sensitive and diagnostically useful immunohistochemical marker of normal endometrial stroma and of endometrial stromal neoplasms. Histopathology.

[8] Abbaszadeh S, Heidari F (2012). Impact of cold ischemia time on outcome of deceased kidney transplantation. Iran J Kidney Dis.

[9] Wieser M, Stadler G, Jennings P, Streubel B, Pfaller W, Ambros P, Riedl C, Katinger H, Grillari J, Grillari-Voglauer R (2008). hTERT alone immortalizes epithelial cells of renal proximal tubules without changing their functional characteristics. Am J Physiol Renal Physiol.

[10] Knafl D, Muller M, Pajenda S, Genc Z, Hecking M, Wagner L (2017). The urine biomarker panel [IGFBP7]x[TIMP-2] (NephroCheck(R) parameter) does not correlate with IGFBP7 and TIMP-2 gene expression in urinary sediment. PLoS One.

[11] Faa G, Gerosa C, Fanni D, Nemolato S, Marinelli V, Locci A, Senes G, Mais V, Van Eyken P, Iacovidou N (2012). CD10 in the developing human kidney: immunoreactivity and possible role in renal embryogenesis. J Matern Fetal Neonatal Med.

[12] Tanigawa S, Nishinakamura R (2016). Expanding nephron progenitors in vitro: a step toward regenerative medicine in nephrology. Kidney Int.

[13] Kaminski MM, Tosic J, Kresbach C, Engel H, Klockenbusch J, Muller AL, Pichler R, Grahammer F, Kretz O, Huber TB (2016). Direct reprogramming of fibroblasts into renal tubular epithelial cells by defined transcription factors. Nat Cell Biol.

[14] Wyatt CM, Dubois N (2017). In vitro generation of renal tubular epithelial cells from fibroblasts: implications for precision and regenerative medicine in nephrology. Kidney Int.

[15] Ali AS, Al-Shraim M, Al-Hakami AM, Jones IM (2015). Epstein- Barr virus: clinical and epidemiological revisits and genetic basis of oncogenesis. The open virology journal.

