# Supplementary material for: Urinary nephrospheres indicate recovery from acute kidney injury in renal allograft recipients – a pilot study
Source: BMC Nephrol. 2019 Jul 9;20:251. doi: 10.1186/s12882-019-1454-3 (PMC6617660; doi:10.1186/s12882-019-1454-3)
Supplement: Supplementary file 1 — Figure S1. Immunohistochemical staining of urinary nephrospheres. Staining for cytokeratin 7 (CK7) was positive for all nephrospheres. The extent of neprilysin (CD10) staining varied strongly among individual cells within different nephrospheres. GATA3 and PAX8 were positive. Figure S2. Quantitative PCR of urinary sediment cells for AQP1 and CASR expression. Relative CASR expression (red bar), AQP1 expression (green bar), and NPHS2 expression (blue bar) in urinary sediment cells. Numbers 2–20 represent urinary cells from non-transplanted patients undergoing AKI; Numbers 21–62 represent urinary cells from transplant recipients undergoing AKI. Values represent fold expression relative to normal healthy kidney tissue (sample 1). (DOCX 4362 kb) [file 12882_2019_1454_MOESM1_ESM.docx]

**Supplementary material**

**Table of contents**

Supplementary figure 1 Page 2

Supplementary figure 2 Page 3

**
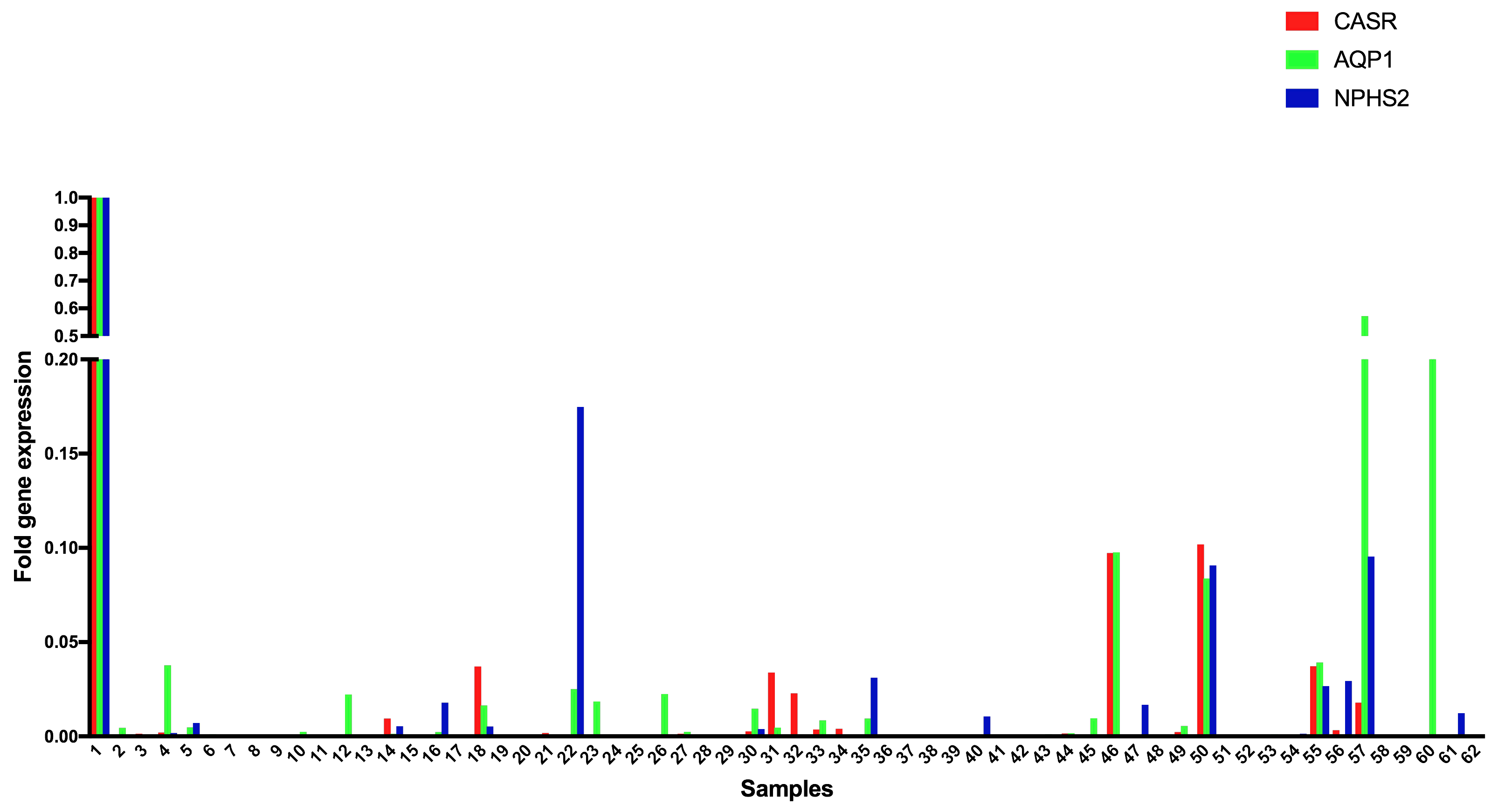
**

**Supplementary Figure 1. Quantitative PCR of urinary sediment cells for AQP1 and CASR expression.** Relative CASR expression (red bar), AQP1 expression (green bar), and NPHS2 expression (blue bar) an in urinary sediment cells. Numbers 2-20 represent urinary cells from non-transplanted patients undergoing AKI; Numbers 21-62 represent urinary cells from transplant recipients undergoing AKI. Values represent fold expression relative to normal healthy kidney tissue (sample 1).

**
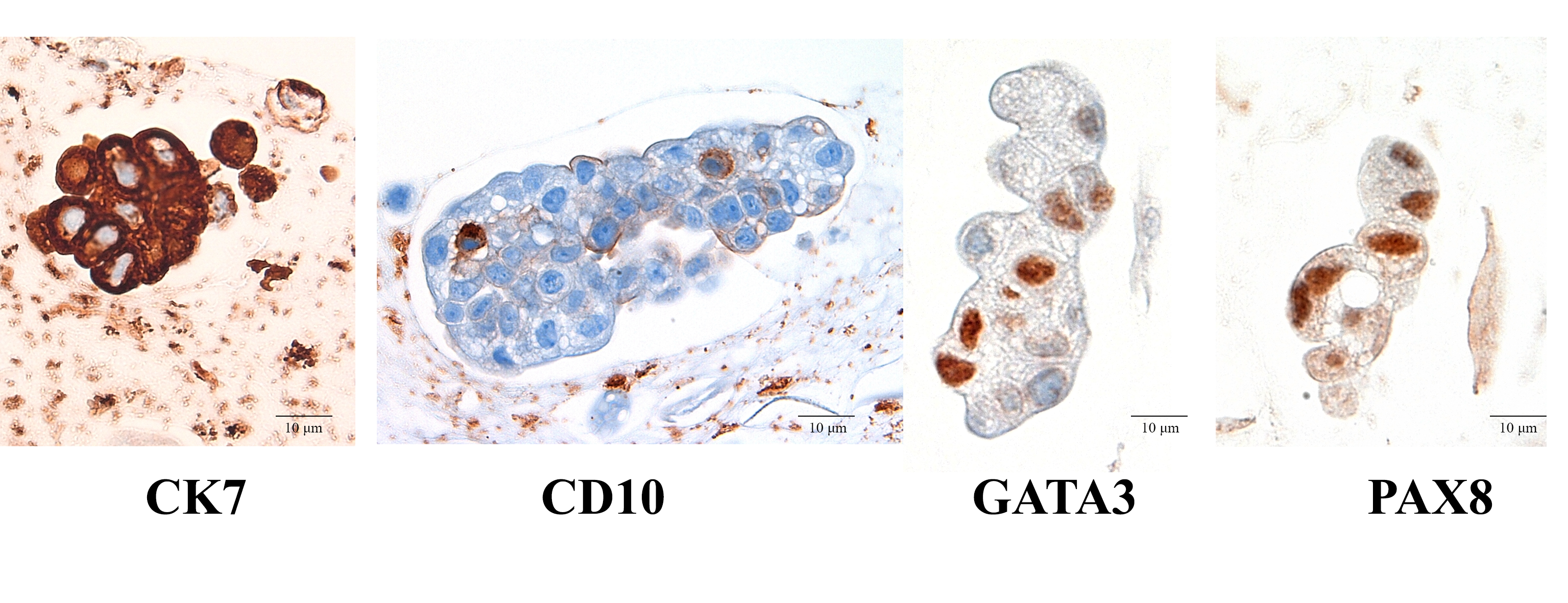
**

**Supplementary Figure 2. Immunohistochemical stainings of urinary nephrospheres.** Staining for cytokeratin 7 (CK7) was positive for all nephrospheres. The extent of neprilysin (CD10) staining varied strongly among individual cells within different nephrospheres. GATA3 and PAX8 were positive.
